# Intestinal Macrophage Autophagy and its Pharmacological Application in Inflammatory Bowel Disease

**DOI:** 10.3389/fphar.2021.803686

**Published:** 2021-11-24

**Authors:** Yang Zheng, Yang Yu, Xu-Feng Chen, Sheng-Lan Yang, Xiao-Long Tang, Zheng-Guo Xiang

**Affiliations:** ^1^ Department of Gastroenterology, 904 Hospital of PLA Joint Logistic Support Force, Wuxi, China; ^2^ Department of Gastroenterology, The Affiliated Suzhou Hospital of Nanjing Medical University, Suzhou, China

**Keywords:** inflammatory bowel disease, macrophage, autophagy, intestinal, inflammation

## Abstract

Inflammatory bowel disease (IBD), comprised of Crohn’s disease (CD) and ulcerative colitis (UC), is a group of chronic inflammatory disorders. IBD is regarded as a severe healthcare problem worldwide, with high morbidity and lethality. So far, despite of numerous studies on this issue, the specific mechanisms of IBD still remain unclarified and ideal treatments are not available for IBD. The intestinal mucosal barrier is vital for maintaining the function of the intestinal self-defensive system. Among all of the components, macrophage is an important one in the intestinal self-defensive system, normally protecting the gut against exotic invasion. However, the over-activation of macrophages in pathological conditions leads to the overwhelming induction of intestinal inflammatory and immune reaction, thus damaging the intestinal functions. Autophagy is an important catabolic mechanism. It has been proven to participate the regulation of various kinds of inflammation- and immune-related disorders via the regulation of inflammation in related cells. Here in this paper, we will review the role and mechanism of intestinal macrophage autophagy in IBD. In addition, several well-studied kinds of agents taking advantage of intestinal macrophage autophagy for the treatment of IBD will also be discussed. We aim to bring novel insights in the development of therapeutic strategies against IBD.

## 1 Introduction

Inflammatory bowel disease (IBD), including Crohn’s disease (CD) and ulcerative colitis (UC), is referred to as a group of idiopathic inflammation-related bowel diseases, which mainly affects the ileum, rectum and colon (Dun et al., 2021; Gallagher et al., 2021; Mazur et al., 2021). The clinical manifestations of IBD are atypical, including diarrhea, abdominal pain, bloody stools and so on (Li et al., 2020; Wang et al., 2020; Yeh et al., 2021). The morbidity of IBD is increasing rapidly in the recent decade, especially in the developed countries, and the diagnosed rate is relatively high in the young and middle-aged people (M’Koma, 2013; Uhlig et al., 2021). So far, the specific mechanisms for the pathogenesis and progression of IBD remain unclarified. According to recent studies, the abnormality of intestinal immune system and inflammatory responses may probably contribute to the onset and development of IBD (Cushing and Higgins, 2021; Nash et al., 2021). Certain environmental, genetic and infectious factors as well as disturbance of intestinal microbiota homeostasis may serve as vital factors to lead to the pathological state intestinal self-protective immune and inflammatory reaction (Owczarek et al., 2016; Lloyd-Price et al., 2019; Bushman et al., 2020; Ryan et al., 2020).

Currently, the intestinal tract is recognized as not only the largest digesting organ as traditionally considered, but also a critical immune organ, since it is frequently exposed and challenged with exogenous invasive microorganisms in the gut lumen (Fiorucci et al., 2021; Lu et al., 2021). As a result, a strong and complete intestinal self-defensive system have been developed in fighting against those damaged factors. In the system, the intestinal mucosal barrier has been recognized to play a vital role in protecting against invasive damage and maintaining the intestinal microbiota homeostasis (Mehandru and Colombel, 2021; Yang and Yu, 2021; You et al., 2021). It is generally revealed that the intestinal microbiota homeostasis mainly includes three layers, including the mucus layer on the surface, epithelium layer and inflammatory and immune cell layer in the submucosa (Grondin et al., 2020; Liu et al., 2020a; Shao et al., 2021). The mucus layer is a gel-like layer containing many kinds of proteins, which cover on the surface of the mucosa to get rid of the direct contacting of epithelium and intestinal lumen contents (Engevik et al., 2019; das Neves et al., 2020). The epithelium layer is referred to as a layer of gut epithelial cells, mainly including intestinal enterocytes, goblet cells, Paneth cells, enteroendocrine cells and so on, functioning in the absorption of nutrition and secretion of certain functional proteins (Shaoul et al., 2005; Haber et al., 2017; Collins et al., 2021). The immune cell layer in the submucosa is comprised of several kinds of immune- and inflammation-related cells, including macrophages, neutrophils, eosinophils, lymphocytes and so on (Perdue and McKay, 1994; Pardigol et al., 1998; Hu et al., 2021).

Among all of those immune- and inflammation-related cells of the immune cell layer in the submucosa, macrophages are one of the most studied ones. It has been shown that the gut holds the largest population of macrophages among all organs. Normally, the majority of tissue macrophages are divided into M1 and M2 subtypes, with M1 subtype displaying pro-inflammatory features and M2 subtype as anti-inflammatory and immunosuppressive characteristics (Ke et al., 2016a; Yunna et al., 2020; Wu et al., 2021a). However, for gut-resident macrophages, they have been reported to hold the characteristics of both M1 and M2 subtypes to produce both pro-inflammatory cytokines including TNF-α and anti-inflammatory cytokines such as IL-10 (Weber et al., 2011; Ke et al., 2016a). Intestinal macrophages have been revealed to play a vital role in the protection of the gut against exotic invasion and damage via the engulfment and presentation of invading antigens to other immune cells for clearance and killing (Vannella and Wynn, 2017; Muller et al., 2020; Viola and Boeckxstaens, 2020). However, the overwhelming activation of such self-protective immune and inflammatory responses induced by macrophages may lead to the disturbance of gut functions and intestinal microbiota homeostasis, thus resulting in certain gut diseases such as IBD (Baumgart and Carding, 2007; Wallace et al., 2014; Fan et al., 2017). Consequently, regulating the abnormality of inflammatory and immune reaction mediated by intestinal macrophages may serve as a potential and promising strategy for the treatment of IBD.

Autophagy is a self-eating catabolic cellular pathway, through which some long-lived proteins or cytoplasmic organelles are degraded and recycled by the integration with lysosomes to maintain cellular homeostasis and normal functions (Wang et al., 2018; Devis-Jauregui et al., 2021; Li et al., 2021). Autophagy has been reported to produce a regulatory effect in various kinds of diseases. In inflammatory and immune diseases, baseline and properly induced autophagy have been proven to effectively alleviate the onset and development of diseases via the suppression of the overwhelming inflammatory and immune reaction (Racanelli et al., 2018; Chimienti et al., 2021; McCormick et al., 2021). Autophagy has also been shown to have close connection with many forms of inflammatory and immune responses in cells, such as the inflammasomes and neutrophil extracellular traps, apoptosis and necrosis (Luo et al., 2021a; Dong et al., 2021; Qin et al., 2021). As a result, uncovering the role and underlying mechanisms of autophagy in inflammation-related disorders is necessary and vital for the development of effective therapeutic strategies. Here in this paper, the role of intestinal macrophage autophagy in IBD as well as its pharmacological applications will be reviewed and discussed based on current related studies. We hope to provide insights in the development of new strategies against IBD.

## 2 Autophagy in Inflammatory Bowel Disease

### 2.1 Biological Features of Autophagy

The word “autophagy” derives from Greet roots “auto” (self) and “phagy” (eating), which means “to eat itself” (Yoshimura et al., 2020; Joffre et al., 2021). Autophagy is a vital cellular catabolic pathway for degrading damaged proteins and organelles to recycle relying on lysosomes (Devis-Jauregui et al., 2021; Wang et al., 2021; Yao et al., 2021). After initially reported in the 1950s, the process of autophagy was systematically uncovered by Dr. Yoshinori Ohsumi, who was awarded to the 2016 Nobel Prize in Medicine or Physiology (Rubinsztein and Frake, 2016; Wollert, 2019). So far, three kinds of classic forms of autophagy have been described, namely microautophagy, macroautophagy and chaperone-mediated autophagy (Dash et al., 2019; Lin et al., 2021; Trelford and Di Guglielmo, 2021). Besides, according to the difference of degrading substrates, several kinds of selective autophagy have also been recognized and reported in various kinds of diseases, including mitophagy, pexophagy, reticulophagy, xenophagy and so on (Kuma et al., 2017; Li et al., 2021; Xu et al., 2021). Since macroautophagy is so far the most studied form of autophagy, here in this paper, the characteristics of macroautophagy and its roles in IBD will be mainly discussed (hereafter referring to “autophagy”).

Over the few decades since the initial description of autophagy, there are more than 30 autophagy-related genes (ATGs) reported to be vital in the process of autophagy (Hu, 2019; Kamel et al., 2020; Zhang et al., 2021). The process of autophagy is generally divided into two steps, including the formation of the autophagosome and integration with the lysosome to form the autolysosome (Wang et al., 2018; Yu et al., 2018; Colletti et al., 2020; Lorincz and Juhasz, 2020; Tan and Chen, 2021). Under the challenge of stressful conditions, inflammation, nutrient insufficiency or ischemia, autophagy is initiated via the triggering of the first step. In the first step, the autophagy-related proteins including ATG13, ATG101, Unc-51-like kinase 1 (ULK1) and focal adhesion kinase family interacting protein of 200 kD (FIP200) are integrated to form the ATG1 complex, which subsequently lead to the assembly of Becline-1, ATG14, VSP15 and VSP34 to form the Class III phosphatidylinositol 3-kinase (PI3K) complex. The activation of Class III PI3K signaling promote the nucleation of membrane to form the single-layer and cup-shaped phagophore. The membrane further expanded and lengthened along with the formation of ATG5-ATG12-ATG16L1 complex and participation of ATG8 [well known as “light chain 3 (LC3)”], which leads to the formation of the double-membrane and cycled autophagosome. The function of autophagosome is to facilitate cargo recruitment and engulfment. In the second step, with the participation of ATG3, ATG4 and ATG7, autophagosome fuses with the lysosome to form the autolysosome, which is regarded as the functional unit of autophagy. In the process of autophagy, Class I PI3K signaling pathway has been revealed as a classic autophagy inhibiting pathway, with the downstream proteins including Class I PI3K, Akt and mammalian target of rapamycin complex 1 (mTORC1) (Yu et al., 2015; Aoki and Fujishita, 2017; Wang et al., 2017).

Recently, the roles of autophagy in various kinds of diseases have been increasingly studied. In central nervous system, it has been demonstrated that autophagy plays a protective role in the cerebral ischemic stroke via the inhibition of neuronal apoptosis in patients and oxygen-glucose deprivation (OGD)-induced models (Han et al., 2018; Ahsan et al., 2021; Tang et al., 2021). In addition, autophagy has been shown to alleviate the severity of symptoms through the suppression of inflammatory reaction in patients with multiple sclerosis and experimental autoimmune encephalomyelitis (EAE) mice models (Hassanpour et al., 2020; Di Rita and Strappazzon, 2021; Yao et al., 2021). In cardiovascular diseases, autophagy has been revealed to alleviate the formation of macrophage-derived foam cells, thus producing a protective effect on the pathogenesis and progression of atherosclerosis and the subsequent myocardial ischemia (Shao et al., 2016; Ouimet et al., 2017; Kumar et al., 2021). So far, the role of autophagy in IBD, especially in intestinal macrophages, has been drawn increasing attention to by researchers. The effects of intestinal macrophage autophagy will be detailly described and discussed based on the reviewing of related previous studies in the following contents.

### 2.2 Intestinal Macrophage Autophagy in Inflammatory Bowel Disease

**FIGURE 1:**
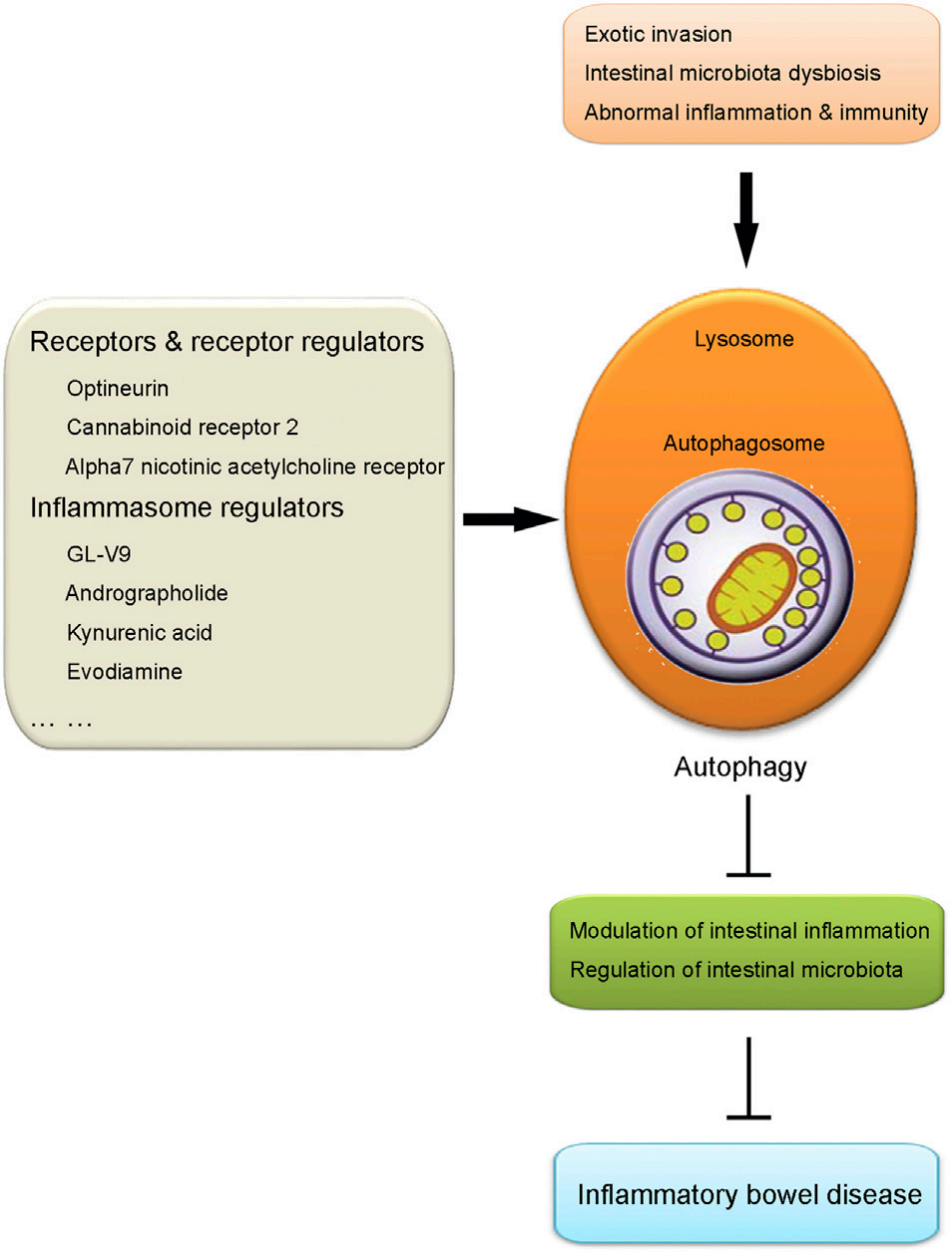
Illustration of the role of intestinal macrophage autophagy in IBD. Under the challenge of certain pathological conditions including exotic invasion, intestinal microbiota dysbiosis and abnormal inflammation and immunity, autophagy process in macrophages is induced, which results in the suppression of intestinal inflammation and regulation of intestinal microbiota. Those effects produced by the induction of macrophage autophagy contribute to the protection against IBD. Several kinds of agents have been proven to alleviate IBD taking advantage of intestinal macrophage autophagy, including receptors and receptor regulators (optineurin, cannabinoid receptor 2, alpha7 nicotinic acetylcholine receptor) and inflammasome regulators (GL-V9, andrographolide, kynurenic acid, evodiamine), etc.

Autophagy has been revealed to have close crosstalk with many inflammatory and immune responses. So far, autophagy in intestinal macrophages has been demonstrated to play a vital role in the regulation of IBD. The mutation of autophagy-related genes has been shown to produce a detrimental effect on the pathogenesis and progression of IBD. In addition, autophagy has also been shown to contribute to modulating intestinal inflammation and maintaining intestinal microbiota homeostasis. Those points of contents will be discussed in this section, respectively (illustrated in Figure 1).

#### 2.2.1 Autophagy-Related Gene Mutation

So far, several autophagy-related genes are vital in the process of intestinal macrophage autophagy (Homer et al., 2010; Parkes, 2012; Saxena et al., 2017; Schwerd et al., 2017). The mutation of such genes, including *Atg16l1*, immunity-related GTPase M (*Irgm*), nucleotide-binding oligomerization domain-containing protein 2 (*Nod2*) and so on, has been reported to contribute to the pathogenesis and progression of IBD. In patients with CD, the existence of polymorphism of *Atg16l1*, *Nod2* and *Irgm* in macrophages has been shown to be the etiology of CD via leading to the deficiency of autophagy (Caprilli et al., 2010). Monocyte-derived macrophages obtained from patients with CD genotyped for *Nod2*, *Atg16l1* and *Irgm* mutations were reported to be unable to restrict adherent-invasive *Escherichia coli* (AIEC) replication and trigger the abnormality in the inflammatory responses influenced by disease activity (Lapaquette et al., 2012; Vazeille et al., 2015). In addition, defective *Nod2* function in intestinal macrophages from CD patients was found to lead to a pro-inflammatory cytokine bias relying on the toll-like receptor 2 (TLR2) signaling (Netea et al., 2004), and loss of wildtype *Nod2* resulted in dysregulated homeostasis of activated fibroblasts and macrophages (Nayar et al., 2021).

In mice models for IBD, it was previously revealed that mice with myeloid *Atg16l1* deficiency largely exacerbated the severity of colitis with the increase of pro-inflammatory cytokine production and decrease of anti-inflammatory cytokine production (Zhang et al., 2017a). The detection of *Atg16l1 T300A* variant in macrophages was also proven as a risk factor for CD, acting as a dominant-negative variant (Zhang et al., 2017a; Gao et al., 2017; Samie et al., 2018). According to a study of single-nucleotide polymorphism (SNP), the SNP for *Atg16l1* (rs2241880, Thr300Ala) was revealed to be strongly associated with the incidence of CD via caspase-3 activation (Luo et al., 2021b). In addition, *Nod2* was reported to mediate an alleviative effect on dextran sulphate sodium (DSS)-induced colitis mice models via suppressing the gram-positive bacteria invasion and damage, while knocking out *Nod2* abolished such effects (Jamontt et al., 2013; Jing et al., 2014; Luo et al., 2021b). For *Irgm*, its loss-of-function mutation was shown to affect the clearance by macrophages of CD-associated AIEC in mice IBD models (Parkes, 2012).

#### 2.2.2 Modulation of Intestinal Inflammation

As discussed previously, the abnormal induction of intestinal inflammatory reaction contributes greatly to the pathogenesis and progression of IBD. Intestinal macrophages act as an important component for the triggering of inflammation and immune reaction. Deficiency of autophagy has been reported to propose an important event in IBD via the enhancement of macrophage-mediated inflammatory reaction (Schwerd et al., 2017; Samoila et al., 2020; Wu et al., 2021b). In patients with IBD, the application of cannabis, a cannabinoid receptor 2 (CB2R) agonist, was shown to alleviate the severity of IBD via the induction of autophagy and suppression of macrophage-mediated inflammation (Tartakover Matalon et al., 2020). Another study conducting on patients revealed that targeting on ATG2B for the inhibition of autophagy by microRNA-143 enhanced the production of macrophage-induced pro-inflammatory cytokines, thus playing a detrimental effect on CD (Lin et al., 2018). High-density lipoprotein (HDL) and apolipoprotein A-I (apoA-I) were reported to suppress the level of intestinal inflammation via autophagy in human intestinal epithelial cell, indicating the intestinal inflammation-suppression effect of autophagy in IBD (Gerster et al., 2015).

In DSS-induced colitis model, it was demonstrated that macrophage-specific V-ATPase subunit ATP6V0D2 significantly alleviated the severity of DSS-induced colitis in mice via restricting the inflammasome activation in macrophages and bacterial infection of the pathogenic *Salmonella typhimurium* (Xia et al., 2019). Those effects were facilitated by the enhancement of autophagosome-lysosome fusion and level of autophagy flux (Xia et al., 2019). In addition, the induction of PTEN-induced putative kinase 1 (PINK1)/Parkin-driven autophagy was shown to protect against DSS-induced colitis through the inactivation of the NLR family pyrin domain containing 3 (NLRP3) inflammasome in macrophages (Mai et al., 2019). A previous study revealed that the induction of AMP-activated protein kinase (AMPK)-induced autophagy could endow the anti-inflammatory properties of intestinal macrophages, thus alleviating DSS-induced colitis (Liu et al., 2020b). In another colitis model induced by trinitro-benzene-sulfonic acid (TNBS), interleukin-33 (IL-33), a well-known anti-inflammatory cytokine, was reported to ameliorate colitis through the enhancement of intestinal macrophage autophagy in the inflammatory gut tissue via regulation of TLR4 signaling pathway (Wang et al., 2019). Furthermore, pharmacological induction of intestinal autophagy via rapamycin, a classic autophagy inducer, was demonstrated to reduce intestinal inflammation from macrophages and improved murine colitis (Macias-Ceja et al., 2017).

#### 2.2.3 Regulation of Intestinal Microbiota

Intestinal microbiota is a vital component of the gut environment (Adak and Khan, 2019; Gomaa, 2020; Jarbrink-Sehgal and Andreasson, 2020). A good intestinal microbiota habitat is necessary for the maintenance of normal gut functions and health for organisms. In normal conditions, four phyla of microbiota are detected to be dominant in the gut of normal human, including *Bacteroidetes*, *Firmicutes*, *Actinobacteria* and *Proteobacteria* (Zhang et al., 2017b; Invernizzi et al., 2021; Mao et al., 2021). However, dysbiosis of intestinal microbiota has been investigated in patients with various digestive disorders, such as colorectal cancer and IBD (Shin et al., 2015; Tomasello et al., 2016; Sun and Shen, 2018; Fan et al., 2021; Sultan et al., 2021). Under certain pathologic conditions, some kinds of harmful microbiota are invaded and largely residue in the gut. Among those harmful microbiota, *Escherichia coli* (*E. coli*) is the most studied one, which belongs to the family of *Enterobacteriaceae* and is usually detected in the lower intestine (Castillo et al., 2005; Rousset et al., 2021). *E. coli*, especially AIEC, have been found to be enriched in the gut of both UC and CD patients (Rolhion and Darfeuille-Michaud, 2007; Mirsepasi-Lauridsen et al., 2019). The assembly and large residue of AIEC have been proven to be harmful for the gut wall via the disturbance of intestinal microbiota homeostasis and triggering of gut inflammatory reaction (Derer et al., 2020; Leccese et al., 2020).

So far, modern studies have revealed a positive role of intestinal macrophage autophagy in fighting against the invasion and damage of AIEC. Dysfunctional autophagy was found to lead to overwhelming inflammatory reaction induced by intestinal macrophages and subsequent gut microbiota dysbiosis in CD (Larabi et al., 2020). In addition, the functional autophagy in macrophages contributes greatly to maintaining the ability of macrophages in recognizing and dealing with the invasion of AIEC (Rolhion and Darfeuille-Michaud, 2007; Caprilli et al., 2010; Buisson et al., 2019). Deficiency of autophagy was shown to lead to the escape of AIEC from macrophages (Caprilli et al., 2010; Palmela et al., 2018; Buisson et al., 2019). Collectively, autophagy is vital to maintain the function of macrophages in dealing with AIEC.

## 3 Applications of Intestinal Macrophage Autophagy in the Treatment of Inflammatory Bowel Disease

As discussed above, intestinal macrophage autophagy plays an important role in the regulation of pathogenesis and progression of IBD. So far, with the efforts of researchers, we are lucky to have several kinds of agents which have been proven to be effective in the alleviation of IBD taking advantage of regulating intestinal macrophage autophagy. Among those agents, two kinds of autophagy regulators, including receptors and receptor regulators, and inflammasome regulators are the most popular and studied ones. As a result, in the following contents, those two kinds of autophagy regulators will be detailly described and discussed, respectively (illustrated in Figure 1 and Table 1).

**TABLE 1:** Pharmacological applications of intestinal macrophage autophagy regulators in the treatment of IBD.

Autophagy regulators	Pharmacological mechanisms related to intestinal macrophage autophagy
Receptors and receptor regulators	
Optineurin	Maintaining pathogen clearance and regulating cytokine production
Cannabinoid receptor 2	Regulating AMPK-mTOR-p70S6K signaling pathway
Alpha7 nicotinic acetylcholine receptor	Inducing the “cholinergic anti-inflammatory pathway” and regulating AMPK-mTOR-p70S6K signaling pathway
Inflammasome regulators	
GL-V9	Activating AMPK signaling
Andrographolide	Downregulating PIK3CA-AKT1-mTOR-RPS6KB1 pathway
Kynurenic acid	Regulating the kynurenic acid/GPR35 axis
Evodiamine	Regulating NF-κB pathway

### 3.1 Receptors and Receptor Regulators

In the recent few years, several kinds of receptors and receptor regulators have been demonstrated to produce an alleviative effect on IBD. For instance, optineurin, a selective autophagy receptor, has been increasing recognized as a critical factor for the maintenance of pathogen clearance and regulation of cytokine production in macrophages (Tschurtschenthaler and Adolph, 2018; Xu et al., 2020). It was reported that the activation of optineurin contributed to the alleviation of IBD, especially CD, via the induction of autophagy, thus suppressing the inflammatory responses mediated by intestinal macrophages (Tschurtschenthaler and Adolph, 2018).

Furthermore, CB2R, a member of the family of G-protein-coupled receptors (GPCR), has been increasingly investigated as an immune and inflammatory modulator (Carbone et al., 2014; Kong et al., 2014; Ke et al., 2016b). Unlike CB1R which is majorly expressed in central nervous system, it is mainly located in the immune system including macrophages and other inflammatory and immune cells in periphery tissues (Carbone et al., 2014; Kong et al., 2014; Ke et al., 2016b). It was demonstrated that the administration of CB2R agonist HU308 significantly alleviated the severity of DSS-induced colitis in mice via the induction of intestinal macrophage autophagy (Ke et al., 2016b). Those protective effects of CB2R agonist on IBD was proven to be mediated by the AMPK-mTOR-p70S6K signaling pathway, a classic autophagy pathway (Ke et al., 2016b).

In addition, alpha7 nicotinic acetylcholine receptor (α7nAChR), a member of the superfamily of cys-loop cationic ligand-gated channels, has been demonstrated to protect against several kinds of inflammation- and immune-related diseases through the triggering of the “cholinergic anti-inflammatory pathway” (Cheng and Yakel, 2015; Qin et al., 2017; Shao et al., 2017; Shao et al., 2019a). Nicotine, an α7nAChR non-selective agonist, has been shown to suppress the production of pro-inflammatory cytokines by macrophages via the microRNA-124/signal transducing activator of transcription (STAT) system in IBD (Qin et al., 2017). A previous study revealed that the administration of PNU282987, a selective α7nAChR agonist, protected against DSS-induced colitis via the induction of AMPK-mTOR-p70S6K signaling-mediated autophagy in intestinal macrophages (Shao et al., 2019a).

### 3.2 Inflammasome Regulators

The inflammasome, a multi-protein oligomer, is widely recognized as a member of innate immunity and also a special form of inflammatory reaction (Mariathasan et al., 2004). Inflammasome is mainly formed in inflammation- and immune-related cells like macrophages, triggering the substantial inflammatory and immune responses through the recognition of pathogen-associated molecular patterns (PAMPs) or danger-associated molecular patterns (DAMPs) (Alexandre et al., 2014; Lei-Leston et al., 2017; Shao et al., 2019b). Inflammasome has been reported to participate in the onset and development of various kinds of inflammation- and immune-related diseases via the production and secretion of certain pro-inflammatory cytokines (Ge et al., 2021; Moretti and Blander, 2021). So far, an increasing number of studies have investigated the role of macrophage inflammasome in IBD, and autophagy has been widely reported to regulate the level of inflammasome in the gut.

According to the recent related studies, several agents have been proven to be effective in the alleviation of IBD taking advantage of inducing autophagy-mediated inflammasome suppression. GL-V9, a small-molecule of AMPK activator, was shown to protect against colitis as well as colitis-associated colorectal cancer through the inhibition of the NLRP3 inflammasome, a well-studied form of inflammasome, by the induction of autophagy in intestinal macrophages (Zhao et al., 2017). Similar effect on colitis and colitis associated colorectal cancer was reported to be produced by another small molecule andrographolide via the downregulation of the PIK3CA-Akt1-mTOR-RPS6KB1 pathway (Guo et al., 2014). In addition, kynurenic acid, an endogenous regulator of social stress-exacerbated colitis, was shown to induce autophagy-dependent degradation of NLRP3 in macrophages through the kynurenic acid/G Protein-Coupled Receptor 35 (GPR35) axis (Zheng et al., 2019). Evodiamine, a chemical component extracted from *Evodiae Fructus*, was shown to suppress the initiation and assembly of the NLRP3 inflammasome via autophagy-mediated regulation of nuclear factor-κB (NF-κB) pathway in macrophages, thus alleviating DSS-induced colitis (Ding et al., 2020).

## 4 Conclusion

In this paper, we have reviewed the role of intestinal macrophage autophagy in the pathogenesis and progression of IBD. We have introduced the biological characteristics of autophagy and discussed the role of intestinal macrophage autophagy in IBD based on reviewing the related studies. In addition, two well-studied kinds of autophagy regulators, including receptors and receptor regulators as well as inflammasome regulators were described. Although the mechanisms of intestinal macrophage autophagy in IBD are increasingly investigated in the recent studies, yet the specific mechanisms remain unclarified. Furthermore, few autophagy inducers have been successfully applied in clinical practice. Consequently, to ultimately take advantage of intestinal macrophage autophagy in the treatment of IBD, further studies are demanded on this issue.
